# Acute Mesenteroaxial Gastric Volvulus Complicated by Splenic Injury Requiring Splenectomy

**DOI:** 10.7759/cureus.117906

**Published:** 2026-10-14

**Authors:** Abenezer S Tedla, Liam Trimble, Chimsimdi Chudi-Nwankwor, Allison R Pescovitz, James O'Connor

**Affiliations:** 1 General Surgery, BronxCare Health System, New York, USA; 2 Surgery, Queens Hospital Center, Queens, USA; 3 General Surgery, Icahn School of Medicine at Mount Sinai, Queens Hospital Center, Queens, USA

**Keywords:** gastric volvulus, gastropexy, mesenteroaxial, splenectomy, splenic injury

## Abstract

Gastric volvulus is a rare but potentially life-threatening surgical emergency associated with significant morbidity and mortality when diagnosis and treatment are delayed. Although complications such as gastric ischemia and perforation are well recognized, splenic injury secondary to gastric volvulus is exceedingly rare. We present the case of a 60-year-old woman who presented with acute epigastric pain, nausea, and vomiting following a meal. Computed tomography demonstrated a mesenteroaxial gastric volvulus. Initial nasogastric tube decompression resulted in symptomatic improvement; however, repeat imaging demonstrated persistent volvulus with increasing free fluid, prompting exploratory laparotomy. Intraoperatively, hemoperitoneum was identified and attributed to splenic capsular tears caused by traction on the gastrosplenic ligament from the distended, volvulized stomach. The patient underwent detorsion of the volvulus, gastropexy, and splenectomy, followed by an uncomplicated recovery in the surgical intensive care unit. This case highlights the importance of maintaining a high index of suspicion for splenic injury in the setting of gastric volvulus and underscores the need for timely surgical intervention when conservative management fails.

## Introduction

Gastric volvulus is defined as an abnormal rotation of the stomach greater than 180 degrees, creating a closed-loop obstruction that may lead to ischemia, necrosis, and perforation [1]. Two primary types are recognized: organoaxial, in which the stomach rotates along its longitudinal axis, and mesenteroaxial, in which the stomach rotates along its transverse axis around a line connecting the lesser and greater curvatures [2]. Mesenteroaxial gastric volvulus involves rotation of the stomach around its short axis, often limited to the mobile pyloric region, leading to gastric outlet obstruction and frequently presenting as an idiopathic or intermittent condition [1]. Acute gastric volvulus carries a mortality rate of up to 30% when complicated by ischemia or perforation [2]. 

Splenic injury as a direct complication of gastric volvulus is exceedingly rare, with only isolated case reports in the literature [3,4]. The close anatomical relationship between the stomach and spleen via the gastrosplenic ligament creates a mechanism by which gastric distension and torsion can transmit tension to the splenic capsule, resulting in laceration and hemorrhage. We present a case of acute mesenteroaxial gastric volvulus complicated by intraoperative discovery of splenic capsular tears and hemoperitoneum, necessitating splenectomy. 

## Case presentation

A 60-year-old woman with a history of hypertension, Graves' disease, and glaucoma presented to the emergency department with acute-onset upper abdominal pain, nausea, and non-bloody, non-bilious emesis of approximately eight hours' duration. Her surgical history included appendectomy, ovarian cystectomy, and anorectal procedures. She denied fever, chills, chest pain, dyspnea, diarrhea, constipation, hematochezia, melena, and urinary symptoms, and reported a normal bowel movement earlier that day. She had experienced no prior similar episodes.

On arrival, blood pressure was 163/97 mmHg, heart rate 84 beats per minute, temperature 36.7°C, respiratory rate 18 breaths per minute, and oxygen saturation 99% on room air. She appeared non-toxic and in no acute distress. The abdomen was soft with upper abdominal tenderness to palpation, without guarding or rebound. The remainder of the examination was unremarkable.

Laboratory studies demonstrated leukopenia (white blood cell count 3.94 × 10³/µL; normal range: 4-11 × 10³/µL), mild anemia (hemoglobin 11.5 g/dL; normal range: 12-16 g/dL), normal platelet count (219 × 10³/µL; normal range: 150-450 x10³/µL), and mildly elevated lactate (1.5 mmol/L; normal range 0.5-1.4 mmol/L). Serum glucose, renal function, electrolytes, and liver function tests were within normal limits (Table 1).

**Table 1:** Laboratory evaluation at presentation.

Laboratory parameter	Patient value	Reference range	Units	Interpretation
WBC count	3.94	4.0-11.0	×10³/µL	Leukopenia
Hemoglobin	11.5	12-16	g/dL	Mild anemia identified on presentation
Platelet	219	150-450	×10³/µL	Normal
Serum lactate	1.5	0.5-1.4	mmol/L	Mild elevation

Contrast-enhanced computed tomography (CT) of the abdomen and pelvis demonstrated abnormal gastric orientation, with the gastric antrum and pylorus positioned anterior to the gastroesophageal junction placing the antrum above the fundus of the stomach, consistent with mesenteroaxial volvulus. The stomach was distended with fluid and gas, with adjacent free fluid and fat stranding in the left upper quadrant. A dilated, gas-filled esophagus indicated antegrade obstruction at the gastroesophageal junction, and swirling of the mesenteric vessels supported the diagnosis. Low attenuation within the posterior spleen was suspicious for infarction. No hiatal hernia was identified (Figure 1).

**Figure 1:**
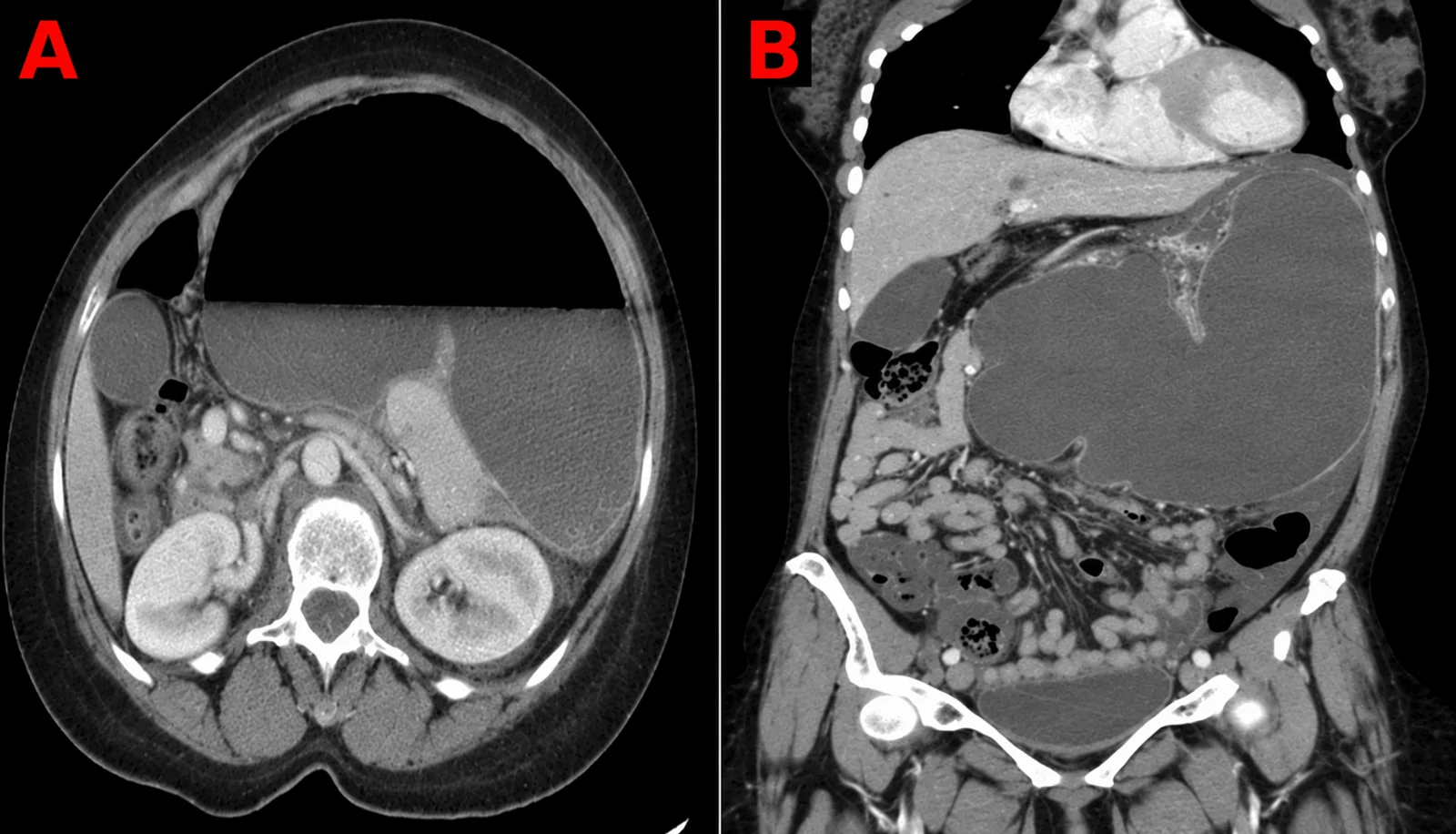
CT imaging at presentation demonstrating gastric volvulus. Axial (A) and coronal (B) images demonstrate marked gastric distension with abnormal positioning and rotation of the stomach, consistent with gastric volvulus.

A nasogastric tube (NGT) was placed without difficulty, with immediate return of 400 mL of gastric content, followed by marked improvement in abdominal distension and epigastric tenderness. She was admitted to the surgical service, made nil per os, and treated with intravenous fluid resuscitation.

Repeat CT of the abdomen and pelvis with oral contrast approximately 12 hours later demonstrated persistent mesenteroaxial volvulus despite appropriate nasogastric tube positioning, with an interval increase in ascites. Given the persistent volvulus and worsening ascites raising concern for evolving gastric ischemia, operative management was discussed with the patient, including the possibility of gastrectomy (Figure 2). After informed consent was obtained, she was taken to the operating room.

**Figure 2:**
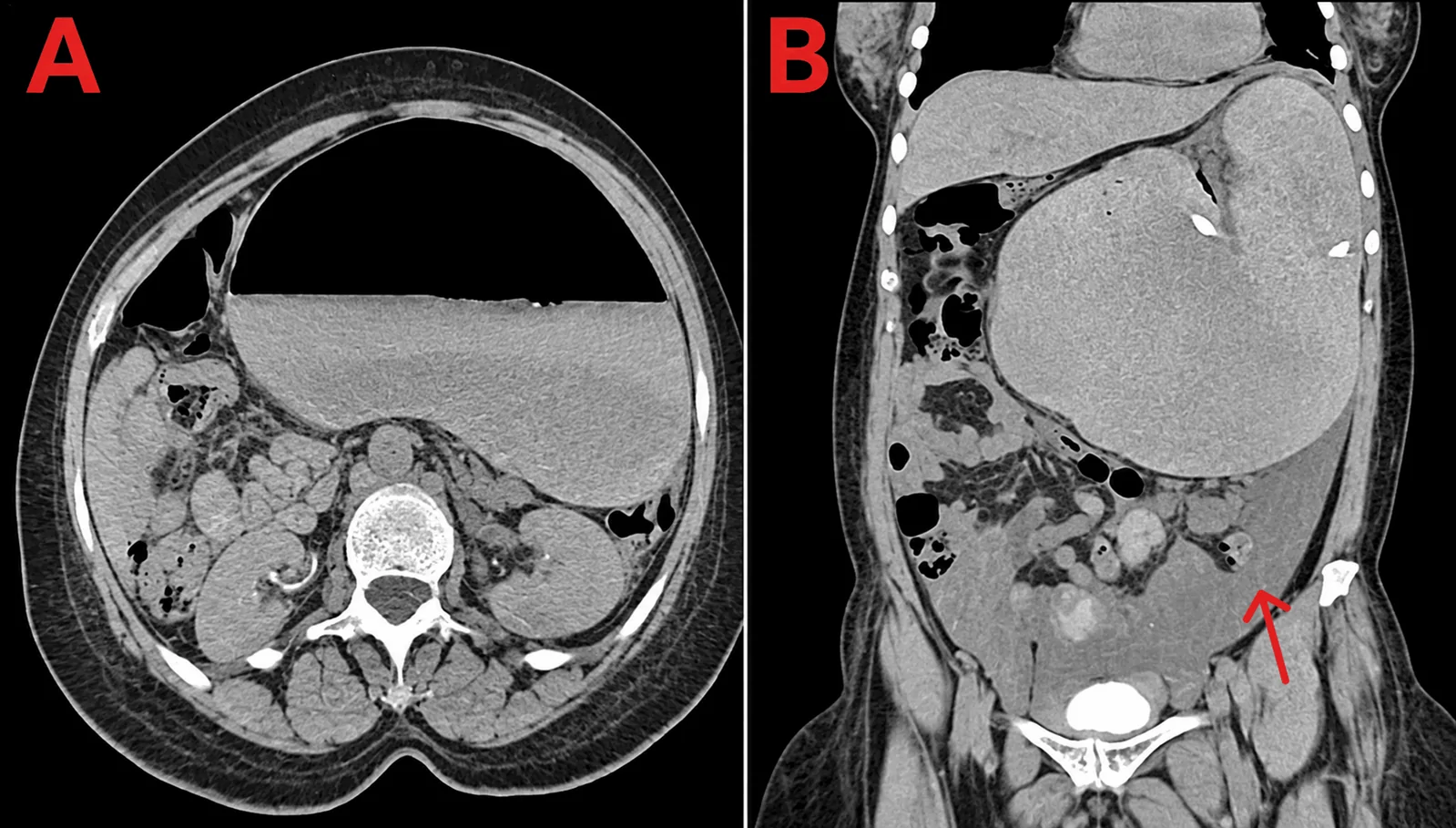
Repeat CT demonstrating gastric volvulus. Axial (A) and coronal (B) CT images demonstrate marked gastric distention with a large air-fluid level and abnormal rotation of the stomach. The arrow indicates an area of worsening ascites.

Abdomen was accessed via midline laparotomy. Upon entering the peritoneal cavity, immediate egress of bright red blood was noted, indicating hemoperitoneum that was present prior to any surgical manipulation. The stomach was markedly distended and protruded through the incision. The distal stomach was found folded cephalad and anteriorly, with the remainder adherent to the omentum and abdominal wall, consistent with mesenteroaxial volvulus. The stomach was delivered, and using the omentum and transverse colon as landmarks, it was untwisted and restored to normal anatomical alignment. The NGT was repositioned, and the stomach was completely decompressed. Importantly, the stomach appeared viable throughout, with no evidence of ischemia or necrosis (Figure 3).

**Figure 3:**
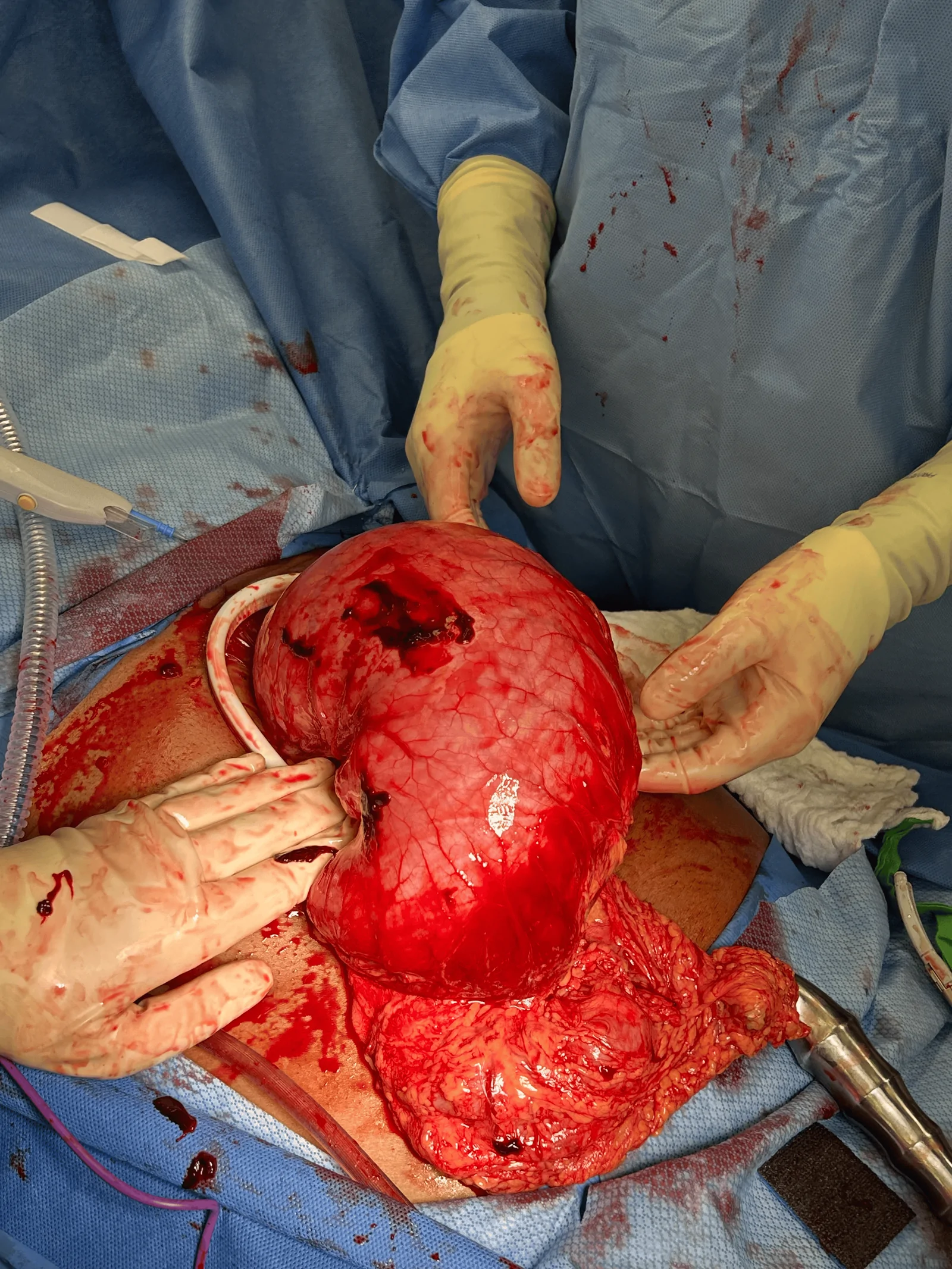
Intraoperative findings of gastric volvulus. The stomach was significantly distended with extensive vascular congestion. A capsular tear of the spleen, attributed to traction from the gastric volvulus, was identified as the source of the associated hemoperitoneum.

Attention was then directed to the source of hemorrhage, which was localized to the left upper quadrant. The lesser sac was opened and found to be free of bleeding. Inspection of the spleen revealed multiple capsular tears inferiorly, attributed to tension on the gastrosplenic ligament from the distended, volvulized stomach. Additionally, a larger intraparenchymal tear was identified lateral to the capsular injuries. Attempts at hemostasis with electrocautery were only partially effective. Given ongoing hemorrhage requiring vasopressor support and blood transfusion, the decision was made to proceed with splenectomy. 

After the splenectomy, the patient's hemodynamics improved. A 10-French flat Jackson-Pratt (JP) drain (Cardinal Health 200, LLC, Dublin, OH) was placed in the left upper quadrant. Anterior gastropexy was performed using two interrupted polydioxanone sutures to fix the stomach to the left abdominal wall. The abdomen was closed in standard fashion. 

The patient was transferred to the surgical intensive care unit for hemodynamic monitoring. She remained hemodynamically stable, and prophylactic piperacillin-tazobactam was continued given the increased risk of infection following splenectomy. On postoperative day 2, a clear liquid diet was initiated and well tolerated. Her diet was subsequently advanced to a regular diet without difficulty. The JP drain was removed, and post-splenectomy vaccinations were administered prior to discharge. She was discharged home in stable condition on postoperative day 6. At a two-week postoperative follow-up visit, she was recovering well without any significant complications.

## Discussion

This case illustrates several important clinical points regarding the diagnosis and management of acute gastric volvulus and its potential complications. 

Gastric volvulus is a rare condition, with acute presentations carrying significant morbidity and mortality [1,2]. The classic Borchardt triad of severe epigastric pain, vomiting, and inability to pass a nasogastric tube is present in as many as 70% of cases [5]. Our patient presented with a relatively benign initial clinical picture, characterized by epigastric pain and vomiting, a benign abdominal examination, and reassuring laboratory findings. Notably, a nasogastric tube was successfully passed, highlighting the absence of the classic Borchardt triad. CT imaging proved essential for diagnosis, consistent with published data demonstrating CT sensitivity approaching 100% for gastric volvulus [2].

In stable patients, acute gastric volvulus can be managed nonoperatively. Initial non-operative measures include nasogastric decompression, while endoscopic therapy may relieve the volvulus through detorsion and, when appropriate, provide gastric fixation with a percutaneous gastrostomy tube [2]. In our patient, initial management with NGT decompression resulted in symptomatic improvement and was used to achieve gastric decompression and temporarily convert an emergent situation into a semi-elective one. However, repeat imaging demonstrated persistent volvulus with worsening ascites, prompting surgical intervention. This approach is consistent with recommendations for prompt operative management in patients who fail conservative therapy [2].

The most notable aspect of this case is the intraoperative finding of hemoperitoneum secondary to splenic capsular and parenchymal tears. This complication has been rarely reported in the literature. Warren et al. described a case of splenic laceration secondary to acute gastric volvulus, attributing the mechanism to the volvulized stomach placing traction on the short gastric vessels [3]. Similarly, Szwerc and Landreneau reported splenic capsular laceration and hemorrhagic shock in the setting of an incarcerated paraesophageal hernia with forceful retching [4]. In our patient, the splenic injuries were likely caused by tension transmitted through the gastrosplenic ligament from the massively distended, volvulized stomach. The abnormal low density in the posterior spleen noted on the initial CT, suspicious for infarct, may have represented early ischemic changes from vascular compromise related to the volvulus. 

The decision to proceed with splenectomy was driven by the extent of splenic injury, ongoing hemorrhage, and hemodynamic instability requiring vasopressors and transfusion. Post-splenectomy management included prophylactic antibiotics and planning for vaccination against encapsulated organisms, consistent with established guidelines for asplenic patients [6]. 

The absence of a hiatal hernia in this case is noteworthy, as most gastric volvulus cases in adults are secondary to paraesophageal hernias or diaphragmatic defects [2]. Primary or idiopathic gastric volvulus, occurring without an associated anatomic defect, accounts for a minority of cases and may be related to laxity of the gastric ligamentous attachments.

## Conclusions

Acute mesenteroaxial gastric volvulus is a rare surgical emergency that may present with nonspecific symptoms mimicking more common gastrointestinal conditions. This case demonstrates that splenic injury can occur as a direct consequence of gastric volvulus due to tension on the gastrosplenic ligament, and clinicians should maintain awareness of this rare but potentially life-threatening complication. Prompt CT imaging is essential for diagnosis, and surgical intervention should not be delayed when conservative management fails to resolve the volvulus. Post-splenectomy patients require appropriate vaccination and long-term infection prophylaxis. 

## References

[1] Carter R, Brewer LA, Hinshaw DB (1980). Acute gastric volvulus. A study of 25 cases. Am J Surg.

[2] Light D, Links D, Griffin M (2016). The threatened stomach: management of the acute gastric volvulus. Surg Endosc.

[3] Warren C, Wallace W, Campbell WJ (2012). Splenic laceration with a twist: a lesson learnt from gastric volvulus. Ann R Coll Surg Engl.

[4] Szwerc MF, Landreneau RJ (2000). Splenic rupture as a consequence of giant paraesophageal hernia. Ann Thorac Surg.

[5] Chau B, Dufel S (2007). Gastric volvulus. Emerg Med J.

[6] Ladhani SN, Fernandes S, Garg M, Borrow R, de Lusignan S, Bolton-Maggs PH (2024). Prevention and treatment of infection in patients with an absent or hypofunctional spleen: a British Society for Haematology guideline. Br J Haematol.

